# Road proximity, air pollution, noise, green space and neurologic disease incidence: a population-based cohort study

**DOI:** 10.1186/s12940-020-0565-4

**Published:** 2020-01-21

**Authors:** Weiran Yuchi, Hind Sbihi, Hugh Davies, Lillian Tamburic, Michael Brauer

**Affiliations:** School of Population and Public Health, Faculty of Medicine, The University of British Columbia, 2206 East Mall, Vancouver, British Columbia V6T 1Z3 Canada

**Keywords:** Road proximity, Air pollution, Greenness, Noise, Neurological disorders, Population-based

## Abstract

**Background:**

Emerging evidence links road proximity and air pollution with cognitive impairment. Joint effects of noise and greenness have not been evaluated. We investigated associations between road proximity and exposures to air pollution, and joint effects of noise and greenness, on non-Alzheimer’s dementia, Parkinson’s and Alzheimer’s disease and multiple sclerosis within a population-based cohort.

**Methods:**

We assembled administrative health database cohorts of 45–84 year old residents (N ~ 678,000) of Metro Vancouver, Canada. Cox proportional hazards models were built to assess associations between exposures and non-Alzheimer’s dementia and Parkinson’s disease. Given reduced case numbers, associations with Alzheimer’s disease and multiple sclerosis were evaluated in nested case-control analyses by conditional logistic regression.

**Results:**

Road proximity was associated with all outcomes (e.g. non-Alzheimer’s dementia hazard ratio: 1.14, [95% confidence interval: 1.07–1.20], for living < 50 m from a major road or < 150 m from a highway). Air pollutants were associated with incidence of Parkinson’s disease and non-Alzheimer’s dementia (e.g. Parkinson’s disease hazard ratios of 1.09 [1.02–1.16], 1.03 [0.97–1.08], 1.12 [1.05–1.20] per interquartile increase in fine particulate matter, Black Carbon, and nitrogen dioxide) but not Alzheimer’s disease or multiple sclerosis. Noise was not associated with any outcomes while associations with greenness suggested protective effects for Parkinson’s disease and non-Alzheimer’s dementia.

**Conclusions:**

Road proximity was associated with incidence of non-Alzheimer’s dementia, Parkinson’s disease, Alzheimer’s disease and multiple sclerosis. This association may be partially mediated by air pollution, whereas noise exposure did not affect associations. There was some evidence of protective effects of greenness.

## Background

Neurological disorders, such as Parkinson’s disease, are one of the leading causes of disability in Canada and in other high income countries [1]. They are associated with a range of adverse impacts that pose daily challenges to patients, their families and health care systems [1, 2]. Estimated costs to the Canadian health care system in 2012 were more than 10 billion dollars [3]. Individuals over 65 years old are expected to comprise approximately 25 % of the Canadian population by 2035 [4, 5]. As neurological disorders display a strong age dependence with incidence peaking at 60–70 years, new cases of neurological disorders along with their economic burden are forecasted to dramatically increase in the next decades [6–8]. Of the neurological disorders, dementia is an umbrella term used to represent a wide range of illnesses that affect the brain and cause progressive decline in cognitive function in adults [9, 10]. Alzheimer’s disease is the most commonly diagnosed type of dementia [11] with previous studies classifying dementia into non-Alzheimer’s dementia and Alzheimer’s disease [12–14].

Despite the population health burden of neurological disorders, modifiable risk factors are largely unidentified, although certain behavioural risks such as smoking and physical activity are suggested to contribute to disease onset [3, 4, 6, 15]. A number of recent studies have focused on environmental exposures such as traffic proximity and air pollution as potential risk factors [16, 17].

Epidemiological studies have reported associations between road proximity and traffic-related air pollution (e.g. nitrogen dioxide) with impaired cognitive function in adults and incidence of neurological disorders [18–21]. Two longitudinal studies in Germany reported that shorter residential distance to roads was positively associated with worse cognitive performance [22, 23]. A large population-based administrative data cohort study in Canada found associations with road proximity and increased incidence of several neurological disorders [18]. Decreased cognitive and central nervous system function from long-term traffic-related air pollution was also reported in cohort studies in the United States [20, 24]. A cross-sectional study in Germany reported a positive association between exposure to traffic-related air pollution and cognitive impairment [21], while weak associations between traffic-related PM_2.5_ and decline in memory performance were found in the United Kingdom [25]. Nitrogen oxides, common markers of traffic-related air pollution, were associated with greater incidence of Alzheimer’s disease in longitudinal studies in Sweden and China [19, 26].

While epidemiological evidence is emerging with respect to the relationships between road proximity, traffic-related air pollution and neurological disorders, additional studies with large populations and considering multiple exposures related to traffic proximity are needed to assess causality. We therefore evaluated associations in population-based linked administrative data cohorts, similar to those previously analyzed with respect to diabetes, cardiovascular and respiratory disease [27–32]. Specifically, we investigated the links between road proximity and air pollution on non-Alzheimer’s dementia (NAD), Alzheimer’s disease (AD), Parkinson’s disease (PD) and multiple sclerosis (MS). As noise and greenness are spatially correlated with road proximity and traffic-related air pollution [33–39] and may also have some impacts on neurological disorders, we evaluated potential joint effects of these environmental exposures on the incidence of these neurological disorders.

## Methods

### Study area and population

Metro Vancouver is a rapidly growing urban area, located in the southwest mainland of the province of British Columbia (BC), Canada. As of 2016, approximately 2.5 million people resided in the metropolitan region, covering 2882 km^2^. Population Data BC provided administrative health data used for creating the study cohort. The Medical Services Plan (MSP) is a mandatory health insurance program in the province of BC, covering nearly all residents [40]. Registration, Physician Visit, and Hospital Discharge data from the MSP were provided by the BC Ministry of Health [41–43]. Vital statistics data were provided by the BC Vital Statistics Agency [44, 45]. The study cohort was established from the MSP central registry and consisted of all adults aged 45–84 years old who resided in Metro Vancouver, were registered with the provincial health insurance plan (MSP), and had lived in Metro Vancouver during the exposure period (January 1994 – December 1998) and follow-up period (January 1999 – December 2003).

Individuals who had a diagnosis of NAD, AD, PD or MS at baseline (January 1999) were excluded. During the exposure period, individual exposures to road proximity, air pollution, noise and greenness were estimated at each person’s residence (residential postal code) using DMTI road network [46], land-use regression models [47–49], a noise prediction model [50] and the satellite-derived Normalized Difference Vegetation Index (NDVI) [51] measure of greenness, respectively. In the urban study area, a postal code generally refers to one side of a block or single multi-unit building. Linkage of the administrative and exposure data were approved by the Behavioural Research Ethics Board of the University of British Columbia (#H05–80442). The linked data allowed for residential history assignment by coding of individuals’ residence locations. Changes of residential postal codes were also accounted for individuals who moved during the study period.

### Case ascertainment

Diagnosis from hospital records, physician visits from MSP [41–45] and prescriptions from PharmaNet (a provincial network linking all pharmacies to a central set of data systems) [52] were applied to identify incident cases of NAD, AD, PD and MS during the 4-year follow-up period. Case definitions of NAD, AD and PD were the same as those applied in a previously published study in Ontario, Canada [18] and based upon published comparisons with primary care physician charts with sensitivity of 78–84% and specificity of 99–100% [53–55].

Specifically, cases of dementia (NAD and AD) were defined as participants having 1) at least one diagnosis at hospital (International Classification of Diseases, ICD, 9th version diagnostic code: 46.1, 290.0–290.4, 294, 331.0, 331.1, 331.5, 331.82 or ICD 10th version diagnostic code F00-F03, G30 after 2002), or 2) three physician claims (code 290, 331) over a two-year period, or 3) a related prescription (e.g., donepezil). Next, cases of AD were selected using code 331 (ICD 9th version) and G30 (ICD 10th version). The remaining cases were NAD. Cases of PD were defined as participants having 1) at least two physician claims (code 332) within a one-year period, or 2) a physician claim and a prescription relating to PD (e.g., Monoamine Oxidase B inhibitors) within 6 months.

Case definition of MS was adopted from that applied in a previously published study in Canada and based upon published comparisons with primary care physician charts with a sensitivity of 80–84% and specificity of approximately 100% [18, 53, 56]. Cases of MS were defined as participants having 1) at least 3 relevant codes (ICD 9th version code 323, 340, 341, 377 or ICD 10th version code G35) for either hospitalization or outpatient visits, 2) at least 1 of the codes was for MS (code 340) or neuromyelitis optica (code 341), and 3) at least one of the 3 codes was for a visit to a neurologist.

### Exposure assessment

#### Road proximity

The CanMap road network (DMTI Spatial, Richmond Hill, ON, Canada) was used to classify roads [46]. Roads that fell in road Category 1 (expressway) and 2 (multi-lane conduits for intracity traffic) were defined as Highways. Road Categories 1 and 2 had 115,000 and 20,000 vehicles per day on average, respectively [57]. Roads that fell in Categories 3 (secondary highways with multiple lanes and large traffic capacity) and 4 (roads for shorter trips within the city) were defined as Major roads. Road Category 3 and 4 had 15,000 and 18,000 vehicles per day on average [57]. For Highways and Major roads, residential proximity to the nearest road was categorized as 1) less than 50 m, or 2) greater than 50 m but less than or equal to 150 m from the postal code centroid as in prior analyses [58].

#### Air pollution

Land-use regression (LUR) models [47–49] specific to Metro Vancouver were applied to estimate exposures to fine particulate matter (PM_2.5_), black carbon, nitrogen dioxide (NO_2_), and nitric oxide (NO). The models were developed based on air pollutant measurements at 116 monitoring sites for NO/NO_2_, 25 monitoring sites for PM_2.5_ and 39 sites for black carbon, along with 55 geospatial variables such as population density, land use and road length. For PM_2.5_, the coefficient of determination (R^2^) of the model was 0.52, including commercial and industrial land use within 300 m, residential land use within 750 m, and elevation. For black carbon, the model (R^2^ = 0.56) included secondary roads within a 100 m buffer, distance to the nearest highway, and industrial land use within 750 m. For NO_2_, the model (R^2^ = 0.56) included major roads within 100 m and 1000 m radius circular buffers, the number of secondary roads within a 100 m buffer, the population density within a 2500 m radius, and elevation. For NO, the model (R^2^ = 0.62) included the same variables and commercial land use within 750 m. Monthly predicted concentrations for each of the air pollutants were generated at each residential postal code (6-digits) during the 1994–1998 exposure period. Based on the residential history of participants in the study, monthly predicted air pollution concentrations were averaged to obtain air pollutant concentrations over the entire exposure period.

Satellite-based PM_2.5_ and NO_2_ from a national land use regression model provided by the Canadian Urban Environmental Health Research Consortium (CANUE) were used in sensitivity analysis [59, 60]. Ground-level PM_2.5_ over North America was estimated by combining 0.01-degree × 0.01-degree resolution optimally estimated Aerosol Optical Depth (AOD) with simulated the aerosol vertical profile and scattering properties. Residual bias in the satellite-derived PM_2.5_ estimates was adjusted using geographically weighted regression (GWR) that incorporated ground measurements [61–63]. The national NO_2_ land use regression model was developed from 2006 National Air Pollution Surveillance (NAPS) monitoring data. The final model (R^2^ = 0.73) included road length within 10 km, 2005–2011 satellite-derived NO_2_ estimates, industrial land use within 2 km, and summer rainfall. Local scale variation was modeled using a deterministic gradient and kernel density measures, which were added to the final model to produce final NO_2_ estimates [61, 64, 65].

#### Noise

A deterministic noise prediction model was used to estimate annual average community noise levels at residential addresses, as described in detail elsewhere [27]. Noise exposures (Annual day-evening-night A-weighted equivalent continuous noise level, L_den_ dB(A)) were calculated based on road type, traffic volume, railway data (e.g., type of train and frequency), flight records, building heights and footprints. Annual weighted noise exposures were generated on a 10 m × 10 m grid, which were averaged for each 6-digit postal code [27]. Noise exposures (L_den_) were weighted towards evening (5-dB(A) weighting) and night (10-dB(A) weighting) noise relative to daytime noise to account for increased noise sensitivity of residents to noise in the evening [30]. Participants’ annual noise exposures were averaged based upon residential history for the exposure period.

#### Greenness

NDVI was used to estimate greenness. All cloud-free images of the Vancouver region from 1999 to 2002 were downloaded from the Landsat Enhanced Thematic Mapper Plus (ETM+) [66]. Average NDVI values in a buffer with 100-m radius surrounding residential addresses were used to calculate yearly greenness values. Annual greenness exposures were averaged based on participants’ residential history for the exposure period (1994–1998). NDVI values range from − 1 to + 1 based on land surface reflectance of visible and near infrared parts of spectrum. NDVI values close to one, indicate higher levels of greenness [51]. While 1999–2002 greenness values were assigned to the 1994–1998 exposure period, greenness exposures are relatively stable over time [67–69].

### Covariates

Individual-level covariates included age, sex and comorbidities that were potentially associated with the outcomes. Using hospital records and MSP at baseline, participants with one of the following conditions were identified: traumatic brain injury, diabetes, hypertension, stroke, coronary heart disease, congestive heart failure, and arrhythmia (ICD codes: 850–854; 250; 401.0, 401.1, 401.9; 434.91; 414.01; 428; 427.9). In addition to age, sex and socioeconomic status, these comorbidities are accepted risk factors for neurodegenerative pathology [18, 70–72].

Neighborhood-level covariates included household income, education (as indicators of socio-economic status) and ethnicity from the 2001 Statistics Canada Census. Because individual socio-economic status (SES) data were not available in this study, SES at the neighborhood-level was used to approximate SES at the individual level [73]. Residential postal codes were used to assign neighborhood (dissemination area) income quintiles to study subjects. For the 2001 Census data, a dissemination area is the smallest census geographic unit and contains 400–600 people. Household size–adjusted average family income was used to rank all dissemination areas, which were divided into quintiles [58]. Education was classified into three levels (without high school diploma, secondary, post-secondary). Ethnicities included Chinese, South Asian and Visible Minorities with strata defined as living in neighborhoods with > 10% of the population of the given ethnic status.

### Statistical analysis

Statistical Analysis Software (SAS version 9.4, SAS Institute Inc., Cary, NC, the United States) [74] was used for data analyses. A cohort study design was applied to NAD and PD and analyzed with Cox proportional hazard models. Cases without hospitalization or death records were treated as censored. Person-years were calculated for study subjects from baseline to the date of the first diagnosis of NAD or PD, or end of follow-up. Household income, education, ethnicity and comorbidity were included as covariates with additional adjustment for age and sex in the Cox proportional hazard models. Relevant model assumptions were examined.

The proportional hazard assumption was not met when applying the Cox proportional hazard model to AD and MS. Further, the number of incident cases of AD and MS was much lower than that of NAD and PD. Therefore, a nested case-control study design was adopted for AD and MS. Each case was matched to 10 controls by age and sex and analyzed by conditional logistic regression. Household income, education, ethnicity and comorbidity were included as covariates and adjusted in the case control analysis.

In both cohort and case-control analysis, we first assessed if road proximity and air pollution were associated with any of the four outcomes, following previously published research conducted in Ontario, Canada [18]. Next, we evaluated relationships with noise and NDVI individually and in joint models with both (road proximity and air pollution) relationships. Further, in the cohort of NAD and PD, sex, ethnicity and age were evaluated in stratified analyses. For AD and MS, ethnicity was evaluated in stratified analysis. In sensitivity analysis, we assessed the relationships between air pollution and the outcomes using alternative national models provided by CANUE, adjusting by the same covariates listed above for each outcome.

## Results

The NAD and PD cohort comprised 7.3 and 7.4 million person-years of observations. During the follow-up period, we identified 13,170 incident cases of NAD, 4201 incident cases of PD, 1277 incident cases of AD and 658 incident cases of MS. Characteristics and exposures at baseline for NAD and PD cohorts are presented in Table 1. In the cohort of NAD, road proximity was moderately correlated with air pollution (e.g. *r* = 0.49 between major road < 50 m or highway < 150 m and black carbon). Greenness was negatively correlated with air pollution (e.g. *r* = − 0.48 between greenness and NO). The air pollution exposure estimates from national models (CANUE) had weak correlations with most of the exposures from local LUR models (r < 0.30). Details of correlations between different exposures and distributions of exposures from CANUE are shown in Additional file 1: Table S1 and S2.

**Table 1:** Descriptive statistics showing covariates and exposures in the cohort of non-Alzheimer’s dementia and Parkinson’s disease, 45–85 years old, 1994–1998

Level	Covariates			Non-Alzheimer’s dementia (*N* = 633949)						Parkinson’s disease (*N* = 634432)							
				Case (*N* = 13170)		Non-case (*N* = 620779)				Case (*N* = 4201)				Non-case (*N* = 630231)			
Individual	Age	Minimum		45		45				45				45			
		Median		76		57				72				58			
		Maximum		83		83				83				83			
		Interquartile		9		18				12				18			
				Count	%	Count		%		Count		%		Count	%		
	Sex	Female		7785	59.1	325967		52.5		1941		46.2		332255	52.7		
		Male		5385	40.9	294812		47.5		2260		53.8		297976	47.3		
	Comorbidity^a^	No		6684	50.7	454399		73.2		2319		55.2		458996	72.8		
		Yes		6486	49.3	166380		26.8		1882		44.8		171235	27.2		
Neighborhood	Household income	Lowest		3426	26.0	113002		18.2		968		23.0		115566	18.4		
		Low		2713	20.6	113505		18.3		857		20.4		115441	18.3		
		Middle		2307	17.5	122122		19.7		772		18.4		123781	19.6		
		Upper middle		2323	17.6	130843		20.1		745		17.7		132509	21.0		
		Upper		2401	18.3	141307		23.7		859		20.5		142934	22.7		
	Education	Less than secondary		1851	14.0	80501		12.9		574		13.7		81883	13.0		
		Secondary		3575	27.2	168156		27.1		1108		26.4		170756	27.1		
		Post-secondary		7744	58.8	372122		60.0		2519		59.9		377592	59.9		
	Chinese	<10% of population in Neighborhood		6325	48.0	295442		47.6		2024		48.2		300035	47.6		
		> 10% of population in Neighborhood		6845	52.0	325337		52.4		2177		51.8		330196	52.4		
	South Asian	<10% of population in Neighborhood		6538	49.7	294834		47.5		2019		48.1		299608	47.5		
		> 10% of population in Neighborhood		6632	50.3	325945		52.5		2182		51.9		330623	52.5		
	Visible minority	<10% of population in Neighborhood		10293	78.2	487142		78.5		3305		78.7		494477	78.5		
		> 10% of population in Neighborhood		2877	21.8	133637		21.5		896		21.3		135754	21.5		
Exposures				Non-Alzheimer’s dementia (*N* = 633949)						Parkinson’s disease (*N* = 634432)							
				Case (*N* = 13170)		Non-case (*N* = 620779)				Case (*N* = 4201)				Non-case (*N* = 630231)			
Road proximity				Count	%	Count		%		Count		%		Count	%		
	Highway <50m	No		12896	97.9	607594		97.9		4104		97.7		279741	97.9		
		Yes		274	2.1	13185		2.1		97		2.3		13375	2.1		
	Highway <150m	No		12417	94.3	586089		94.4		3961		94.3		595005	94.4		
		Yes		753	5.7	34690		5.6		240		5.7		35226	5.6		
	Major road <50m	No		12112	91.9	575818		92.8		3878		92.3		584493	92.7		
		Yes		1058	8.1	44961		7.2		323		7.7		45738	7.3		
	Major road <50m or Highway <150m	No		11472	87.1	546563		88.0		3679		87.6		554783	88.0		
		Yes		1698	12.9	74216		12.0		522		12.4		75448	12.0		
Air pollution		Minimum	Medium	Maximum	Interquartile	Minimum	Medium	Maximum	Interquartile	Minimum	Medium	Maximum	Interquartile	Minimum	Medium	Maximum	Interquartile
	PM_2.5_ (μg/m³)	0.00	4.1	10.3	1.5	0.00	4.0	10.6	1.6	0.1	4.1	10.4	1.7	0.0	4.0	10.6	1.6
	Black carbon (μg/m³)	0.00	1.1	5.02	1.2	0.00	0.8	5.6	1.0	0.0	1.1	5.0	1.3	0.0	1.0	5.6	1.0
	NO_2_ (ppb)	12.0	31.1	61.4	9.2	10.9	29.9	66.6	9.0	12.0	31.3	57.7	9.2	10.9	29.9	66.6	9.0
	NO (ppb)	6.8	29.5	119.3	12.1	6.6	28.4	176.6	13.6	8.2	29.7	101.0	13.7	6.6	28.4	176.6	13.6
Noise (L_den_ dB(A))		33.0	60.8	86.5	6.0	4.4	60.7	92.4	5.5	35.4	60.8	79.7	5.6	4.4	60.7	92.4	5.5
Greenness (NDVI)		-0.1	0.2	0.6	0.1	-0.1	0.3	0.6	0.1	-0.0	0.3	0.6	0.1	-0.1	0.3	0.6	0.1

^a^Comorbidities include traumatic brain injury, diabetes, hypertension, stroke, coronary heart disease, congestive heart failure and arrhythmia

**Table 2:** Descriptive statistics showing covariates and exposures in the analysis of Alzheimer’s disease and Multiple sclerosis, 45-84 years old, 1994-1998

Level	Covariates			Alzheimer’s disease (*N*= 13498)						Multiple Sclerosis (*N*=7232)							
				Case (*N*= 1227)		Control (*N* = 12271)				Case (*N*= 658)				Control (*N* = 6574)			
				Count	%	Count		%		Count		%		Count		%	
Individual	Comorbidity^a^	No		642	52.3	7164		58.4		527		80.1		5300		80.6	
		Yes		585	47.7	5107		41.6		131		19.9		1274		19.4	
Neighborhood	Household income	Lowest		312	25.4	2948		24.0		113		17.2		1071		16.3	
		Low		238	19.4	2368		19.3		136		20.7		1166		17.6	
		Middle		200	16.3	2275		18.6		130		19.8		1293		19.7	
		Upper middle		253	20.6	2235		18.2		140		21.2		1483		22.6	
		Upper		224	18.3	2445		19.9		139		21.1		1561		23.8	
	Education	Less than secondary		275	22.4	2886		23.5		130		20.8		1415		21.5	
		Secondary		327	26.7	3314		27.0		159		24.2		1755		26.7	
		Post-secondary		625	50.9	6071		49.5		369		55.0		3404		51.8	
	Chinese	<10% of population in Neighborhood		699	57.0	5890		48.0		337		51.2		3147		47.9	
		> 10% of population in Neighborhood		528	43.0	6381		52.0		321		48.3		3427		52.1	
	South Asian	<10% of population in Neighborhood		513	41.8	6125		49.9		329		50.0		3030		46.1	
		> 10% of population in Neighborhood		714	58.2	6146		50.1		329		50.0		3544		53.9	
	Visible minority	<10% of population in Neighborhood		959	78.2	9625		78.4		518		78.7		5138		78.2	
		> 10% of population in Neighborhood		268	21.8	2646		21.6		140		21.3		1436		21.8	
Exposures				Alzheimer’s disease (*N*= 13498)						Multiple Sclerosis (*N*=7232)							
				Case (*N*= 1227)		Control (*N* = 12271)				Case (*N*= 658)				Control (*N* = 6574)			
Road proximity				Count	%	Count		%		Count		%		Count		%	
	Highway <50m	No		1203	98.1	12064		98.3		640		97.3		6434		97.9	
		Yes		24	1.9	207		1.7		18		2.7		140		2.1	
	Highway <150m	No		1169	95.3	11728		95.5		615		93.5		6194		94.2	
		Yes		58	4.7	545		4.5		43		6.5		380		5.8	
	Major road <50m	No		1134	92.4	11543		94.1		591		89.8		6082		92.5	
		Yes		93	7.6	728		5.9		67		10.2		492		7.5	
	Major road <50m or Highway <150m	No		1082	88.2	11060		90.1		556		84.5		5759		87.6	
		Yes		145	11.8	1211		9.9		102		15.5		815		12.4	
Air pollution		Minimum	Medium	Maximum	Interquartile	Minimum	Medium	Maximum	Interquartile	Minimum	Medium	Maximum	Interquartile	Minimum	Medium	Maximum	Interquartile
	PM_2.5_ (μg/m³)	0.7	4.0	10.2	1.5	0.0	4.0	10.2	1.5	0.6	4.1	10.2	1.5	0.0	3.9	10.4	1.7
	Black carbon (μg/m³)	0.0	1.0	5.0	1.2	0.0	1.0	5.0	1.1	0.0	1.0	5.0	1.3	0.0	1.0	5.0	0.9
	NO_2_ (ppb)	16.4	28.5	57.5	8.2	12.2	30.5	62.4	9.0	14.4	30.1	57.6	8.0	11.8	29.6	58.0	8.8
	NO (ppb)	13.2	27.5	108.1	10.4	6.8	29.2	126.0	13.9	11.0	29.6	71.3	14.5	7.1	28.1	108.9	13.4
Noise (L_den_ dB(A))		44.5	60.6	77.2	5.6	32.2	60.7	85.8	5.8	36.2	60.3	82.5	5.4	33.4	60.6	84.4	5.6
Greenness (NDVI)		-0.0	0.3	0.6	0.1	-0.1	0.3	0.6	0.1	0.0	0.3	0.5	0.1	-0.1	0.3	0.6	0.1

^a^Comorbidities include traumatic brain injury, diabetes, hypertension, stroke, coronary heart disease, congestive heart failure and arrhythmia

For NAD, the median age (76 years) of cases was older than that of non-cases (57 years) with a corresponding much higher percentage of comorbidity in cases (49% vs 26%). All other measured characteristics were similar between cases and non-cases (Table 1). Among cases of NAD, the median PM_2.5_ concentration during the exposure period was 4.1 μg/m^3^ with an interquartile range (IQR) of 1.5 μg/m^3^. The median black carbon concentration was 1.0 μg/m^3^ (IQR: 1.2 μg/m^3^) and median concentrations of NO_2_ and NO were 31.0 ppb (IQR: 9.1 ppb) and 29.5 ppb (IQR: 12.0 ppb), respectively. Distributions of the exposures in individuals without NAD were similar to those of cases (Table 1).

Similarly, PD cases were much older (median age 72 vs 58 years old) and with a higher proportion of comorbidity (44% vs 27%) compared to non-cases. The remaining characteristics were similar in cases and non-cases (Table 1). Distributions of the exposures in individuals without PD were similar with those of cases (Table 1) and those described above for NAD. Details of descriptive statistics for Alzheimer’s disease and Multiple sclerosis are presented in Table 2.

Based on the magnitude and patterns of effect estimates with non-symmetric confidence intervals around 1, living near major roads or a highway was associated with increased hazard ratios for both NAD and PD (Additional file 1: Figure S1; Table 3). The effects of road proximity for both outcomes were attenuated after accounting for greenness, with reductions (0.3–6.2%) in hazard ratios observed in all proximity categories. Similar increasing patterns with respect to odds ratios (OR) associated with road proximity were observed for AD and MS with large attenuation (11–28%) of the AD association by greenness. Greenness did not attenuate the OR for MS and there were with some indications of increased MS odds ratios after accounting for greenness (Additional file 1: Figure S2).

**Table 3:** Hazard and odds ratios (per one Interquartile range for whole population) between exposures and Non-Alzheimer’s dementia, Parkinson’s disease, Alzheimer’s disease and Multiple sclerosis

Exposures	Non-Alzheimer’s dementia*	Parkinson’s disease*	Alzheimer’s disease**	Multiple sclerosis**
	Hazard ratio (95% CI) per Interquartile Range [IQR]	Hazard ratio (95% CI) per Interquartile Range [IQR]	Odds ratio (95% CI) per Interquartile Range [IQR]	Odds ratio (95% CI) per Interquartile Range [IQR]
Road proximity				
Highway < 50 m	1.03 (0.91, 1.16)	1.12 (0.91, 1.38)	1.19 (0.74, 1.91)	1.20 (0.69, 2.09)
Highway < 50 m + Greenness	1.02 (0.87, 1.20)	1.12 (0.85, 1.46)	0.85 (0.42, 1.74)	1.09 (0.52, 2.29)
Highway < 150 m	1.10 (1.02, 1.19)	1.06 (0.93, 1.22)	1.03 (0.75, 1.41)	0.97 (0.68, 1.39)
Highway < 150 m + Greenness	1.06 (0.96, 1.17)	1.02 (0.86, 1.21)	0.81 (0.53, 1.24)	1.05 (0.66, 1.67)
Major road < 50 m	1.15 (1.08, 1.23)	1.09 (0.96, 1.23)	1.26 (0.96, 1.64)	1.45 (1.06, 1.97)
Major road < 50 m + Greenness	1.15 (1.07, 1.24)	1.02 (0.89, 1.18)	1.12 (0.81, 1.54)	1.75 (1.19, 2.54)
Major road < 50 m or Highway < 150 m	1.14 (1.07, 1.20)	1.07 (0.96, 1.18)	1.19 (0.95, 1.49)	1.25 (0.96, 1.63)
Major road < 50 m or Highway < 150 m + Greenness	1.12 (1.05, 1.20)	0.99 (0.88, 1.13)	1.02 (0.77, 1.36)	1.50 (1.08, 2.08)
Air pollution				
PM_2.5_ (μg/m^3^)	1.02 (0.98, 1.05) [1.54]	1.09 (1.02, 1.16) [1.65]	0.90 (0.76, 1.07) [1.54]	1.25 (0.93, 1.70) [1.72]
PM_2.5_ (μg/m^3^) + Noise	1.02 (0.98, 1.05)	1.08 (1.01, 1.15)	0.91 (0.76, 1.09)	1.40 (0.98, 1.97)
PM_2.5_ (μg/m^3^) + Greenness	1.02 (0.98, 1.05)	1.08 (1.01, 1.15)	0.95 (0.80, 1.13)	1.43 (1.04, 1.97)
Black carbon (μg/m^3^)	1.01 (0.98, 1.04) [1.06]	1.03 (0.97, 1.08) [1.02]	1.02 (0.88, 1.18) [1.09]	0.93 (0.75, 1.15) [0.90]
Black carbon (μg/m^3^) + Noise	1.01 (0.98, 1.04)	1.02 (0.96, 1.09)	0.91 (0.76, 1.08)	0.95 (0.74, 1.23)
Black carbon (μg/m^3^) + Greenness	1.01 (0.98, 1.04)	1.01 (0.96, 1.07)	1.05 (0.90, 1.22)	0.97 (0.78, 1.21)
NO_2_ (ppb)	1.02 (0.99, 1.06) [9.06]	1.12 (1.05, 1.20) [9.03]	0.84 (0.70, 0.99) [8.96]	1.02 (0.78, 1.44) [8.70]
NO_2_ (ppb) + Noise	1.01 (0.97, 1.06)	1.09 (1.01, 1.18)	0.77 (0.63, 0.95)	1.06 (0.69, 1.62)
NO_2_ (ppb) + Greenness	1.02 (0.99, 1.06)	1.10 (1.03, 1.19)	0.87 (0.72, 0.15)	1.16 (0.80, 1.68)
NO (ppb)	1.00 (0.96, 1.04) [13.47]	1.03 (0.96, 1.11) [13.59]	0.91 (0.77, 1.11) [13.68]	0.85 (0.62, 1.16) [13.41]
NO (ppb) + Noise	0.99 (0.95, 1.04)	1.01 (0.93, 1.11)	0.84 (0.67, 1.05)	0.88 (0.59, 1.29)
NO (ppb) + Greenness	0.99 (0.95, 1.04)	0.99 (0.92, 1.08)	0.98 (0.80, 1.20)	0.93 (0.66, 1.29)
Noise (L_den_ dB(A))	1.01 (0.99, 1.04) [5.53]	1.01 (0.97, 1.05) [5.54]	0.99 (0.92, 1.08) [5.75]	0.92 (0.82, 1.03) [5.50]
Greenness (NDVI)	0.95 (0.92, 0.97) [0.11]	0.97 (0.93, 1.01) [0.11]	1.24 (1.13, 1.35) [0.12]	1.14 (1.00, 1.30) [0.12]

*Covariates included for Non-Alzheimer’s dementia and Parkinson’s disease: Age, sex, comorbidities, household income, education and ethnicity; **Covariates included for Alzheimer’s disease and Multiple sclerosis: comorbidities, household income, education and ethnicity

**Fig. 1:**
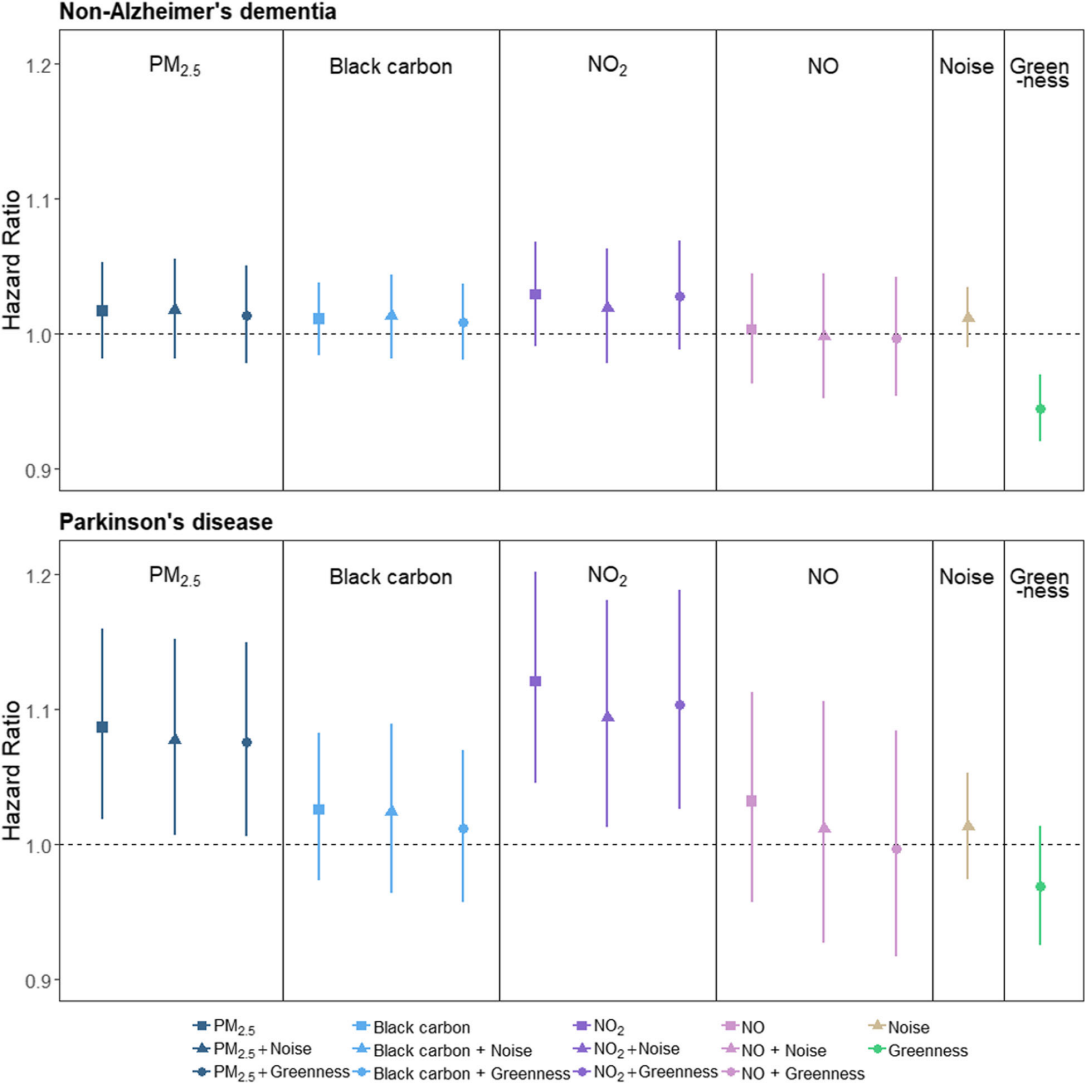
Hazard ratios (95% confidence interval) associated with air pollution, noise and greenness (per Interquartile range as indicated in Table 3) for non-Alzheimer’s dementia and Parkinson’s disease. PM_2.5_ = fine particulate matter, NO_2_ = Nitrogen dioxide, NO = Nitric oxide

Air pollutants, except for NO, were generally associated with slightly increased hazard ratios (HR) for both NAD and PD (e.g. HR for NAD = 1.02, 95% CI: 0.98–1.06 per IQR increase in NO_2_). While there were small effects of noise on both outcomes, including noise in models had essentially no impacts on air pollutant HRs. In single exposure models, greenness was associated with a lower HR for developing NAD (HR: 0.94, 95% CI: 0.92, 0.97) and PD (HR: 0.96, 95% CI: 0.92, 1.01) and when included in models with air pollutants, greenness attenuated the effects of air pollution on both NAD and PD. The protective effect of greenness was more evident for PD compared to NAD (Fig. 1, Table 3).

Specifically, for PM_2.5_ we observed increased HRs for NAD, PD and MS. Elevated HRs were also observed for BC and NO_2_ for NAD and PD, but not for MS. Associations between air pollutants, AD and MS were generally null with wide confidence intervals. There was no evidence of primary effects of noise, while greenness was associated with increased ORs for both AD and MS (Additional file 1: Figure S3). In sensitivity analysis, no patterns of increasing effect estimates were observed between air pollution estimates from CANUE and any of the outcomes.

We did not observe consistent patterns of increased risk by sex, although for PD and NAD with air pollutants hazard ratios were somewhat higher for females. Individuals living in neighborhoods where > 10% of the population was Chinese had higher incidence of NAD when they lived near a major road or a highway compared to those in neighborhoods with < 10% Chinese residents. Both road proximity and air pollution had greater effects on incidence of NAD and PD among people aged under 65. Individuals living in areas with > 10% visible minorities who lived near major roads had a much greater risk of developing AD and MS than individuals living in areas with < 10% visible minorities (Additional file 1: Table S3-S5).

## Discussion

We observed increasing patterns of associations between road proximity across subcategories (e.g. Major road < 50 m, Major road < 50 m or Highway < 150 m, etc.) with hazard and odds of developing NAD, PD, AD and MS. PM_2.5_, black carbon and NO_2_ were associated with increased incidence of NAD and PD. While noise did not affect any of the associations with air pollution, there were indications that greenness was protective for the development of NAD, PD and AD. Overall, we saw indications of associations between air pollution with NAD and PD, but in general not with AD or MS. We did observe increased OR for MS in association with PM_2.5_, but not for any of the other air pollutants. We found associations for air pollution from locally developed LUR models, while sensitivity analysis using air pollution exposure estimated in national exposure models showed no associations. One possible reason was that observed associations related to air pollution estimated from LUR models describe microscale variations in which traffic was a major source and which are not as well-characterized by the national scale models. Greenness was associated with higher incidence of both AD and MS. While this may indicate a harmful effect of greenness proximity on these outcomes, this finding may also result from residual confounding due to unmeasured and/or unidentified spatially-varying risk factors for AD and MS. Overall, we did not find associations with AD or MS for any exposures besides road proximity.

This analysis was the first large population-based study to investigate the onset of four major neurological disorders in association with road proximity and air pollution as well as joint effects of noise and greenness. To our knowledge, only three previous studies describing relationships between air pollution and neurological disorders were population-based [18, 75, 76] with sample sizes ranging from 95,000–4.4 million (e.g. number of cases of PD was about 30,000 in a Canadian population-based study with a sample size of 2.2 million). Our results were mostly consistent with previous population-based studies which reported positive associations between road proximity and neurological disorders. For example, a large population-based cohort study using similar measures of traffic proximity in Canada found that living close to heavy traffic was associated with a 7% increase in the hazard of developing dementia, compared to our estimates of 3–15% increases, depending on the type of road. While we found associations between road proximity and all four neurological disorders, the previous study did not observe associations between road proximity, with PD or MS [18].

Our results with respect to associations between air pollution (from LUR models), NAD, PD, and AD were consistent with several previous studies. Large-scale multi-city studies in the United States found that for every 1 μg/m^3^ increase in annual PM_2.5_, risk of NAD and PD increased by 1.3 to 8% [77, 78], compared to our estimate of a 9% increase per an interquartile range increase in PM_2.5_ of 1.5 μg/m^3^. A matched case-control study in Denmark found 9% risk increase in PD for one interquartile range increase (2.97 μg/m^3^) in NO_2_ [79], somewhat smaller than our estimate of a 12% increase for an interquartile range increase in NO_2_ of ppb ( ~17 µg/m^3^). A cohort study in China reported 9% higher risk for AD for 1 μg/m^3^ increase in PM_2.5_ [76], whereas our findings for AD were largely null. However, a recent study in Netherlands reported that there was no positive relationship between ambient air pollution and Parkinson’s disease [80].

Results indicating that including only noise in models did not modify any of the associations between air pollution and adult neurologic diseases were also consistent with previous studies. A recent population-based longitudinal study on memory and aging in Sweden and a retrospective cohort study in England found no evidence that exposure to noise contributed to the risk of dementia in combination with air pollution [81, 82]. These indications of null effects of noise on adult neurologic diseases differ from studies of children where consistent associations with cognitive impairments have been reported [83]. As has been shown for other outcomes such as mental health and all-cause mortality [36, 84–87], we found that greenness had some protective effects and attenuated effects of road proximity on NAD, PD, AD and MS. Greenness also attenuated effects of air pollution on NAD and PD. In single exposure models, greenness was associated with lower hazards of developing NAD and PD. These results indicate potential beneficial effects of green space [87] and the importance of accounting for potential joint effects of greenness to reveal unbiased associations between road proximity, air pollution and neurological disorders.

Our study has several strengths. First, while we could not account for all potential risk factors in this large population-based cohort, we included neighborhood-level household income and education in the models but did not have individual-level information on education, income or behavioural risk factors. Further, the study population included essentially all adults within the study region. Case ascertainment was based on validated algorithms with high sensitivity and specificity [53–55]. In addition, because of the availability of detailed information on medical and residential history, we were able to account for changes in addresses and comorbidities which may be associated with both exposures and outcomes.

Despite these strengths, our study has several limitations. First, due to data availability we were unable to account for certain lifestyle or behavioral risk factors (e.g. smoking, physical activity, etc.) [88, 89]. Second, exposures to air pollution, noise and greenness were assessed based on residential postal codes, which may lead to some exposure misclassification. As is common in such large cohort analyses, these exposure estimates did not fully represent personal exposures as they did not account for individual factors (time spent at home and travelling patterns etc.) or exposures encountered indoors. Third, the analyses were focused on the 1999–2003 period and may not accurately reflect current exposures, disease management and progression or the role of other risk factors. Fourth, the issue of under-representation exists. While cases were ascertained based on criteria with very high specificity and relatively high (78–84%) sensitivity compared to physician diagnosis, not all incident cases were captured in our study. Fifth, the exposure period was relatively short (1994–1998), in contrast to the longer period during which PD or AD may develop. Given that exposures before 1994 were not available, this limitation may lead to non-differential exposure misclassification and bias towards the null. While we did account for changes in address during the exposure period, during the period of disease progression, people may choose to move in order to accommodate the disabling conditions of their diseases. Therefore, the exposures that we assessed may only include those exposures occurring after changes in residence to accommodate worsening of disease, but not include exposures that contribute to the onset of the disease. Last, as the postal code data alone do not allow us to identify types of residences (e.g. high-rise, single house), we were unable evaluate the potential influence of vertical gradients in distance or pollution. However, a study in Hong Kong reported that including vertical gradient information did not lead to meaningful differences or changes in estimates of effect for the association between air pollution with mortality [90].

## Conclusions

In this large population-based study, living near roads was linked with higher incidence of NAD, PD, AD and MS. Although results were not entirely consistent, air pollution was linked with NAD and PD, but not AD or MS. Greenness was found to have some protective effects while impacts of noise were generally null. Given the high proportion of the population living in proximity to traffic, the spatial correlations with proximity, several environmental factors and the growing prevalence of neurological disorders, future studies in other urban areas which address potential joint effects of multiple environmental exposures are warranted.

## Supplementary information

**Additional file 1: Table S1.** Correlations between road proximity, air pollution, noise and greenness in cohorts of Non-Alzheimer’s dementia, Parkinson’s disease, Alzheimer’s disease and Multiple sclerosis. **Table S2.** Distributions of exposures from Canadian Urban Environmental Health Research Consortium (CANUE). **Table S3.** Hazard ratios between exposures and Non-Alzheimer’s dementia stratified by sex (Males, Females), ethnicity (Chinese, South Asian, Visible Minority) and age (> = 65 years, < 65 years). **Table S4.** Hazard ratios between exposures and Parkinson’s disease stratified by sex (Males, Females), ethnicity (Chinese, South Asian, Visible Minority) and age (> = 65 years, < 65 years). **Table S5.** Odds ratios between exposures and Alzheimer’s disease and Multiple sclerosis stratified by ethnicity (Chinese, South Asian, Visible Minority). **Figure S1.** Hazard ratios (95% confidence interval) associated with road proximity for non-Alzheimer’s disease and Parkinson’s disease. HW = Highway, MR = Major road. **Figure S2.** Odds ratios (95% confidence interval) associated with road proximity for Alzheimer’s disease and Multiple sclerosis. HW = Highway, MR = Major road. **Figure S3.** Odds ratios (95% confidence interval) associated with air pollution, noise and greenness (per Interquartile range as indicated in Table 3) for Alzheimer’s disease and Multiple sclerosis. PM_2.5_ = fine particulate matter, NO_2_ = Nitrogen dioxide, NO = Nitric oxide.

## Data Availability

The data that support the findings of this study are available from the Medical Services Plan of British Columbia and other Data Stewards but restrictions apply to the availability of these data, which were used under agreement for the current study, and so are not openly available. Data are however accessible via Population Data BC and the relevant Data Stewards following approval of a Data Access Request and within the terms of Population Data BC access.

## Abbreviations

AD: Alzheimer’s disease
AOD: Aerosol Optical Depth
BC: British Columbia
CANUE: Canadian Urban Environmental Health Research Consortium
dB(A): A-weighted decibels
ETM+: Enhanced Thematic Mapper Plus
GWR: Geographically weighted regression
HR: Hazard ratio
ICD: International Classification of Diseases
IQR: Interquartile range
L_den_ dB(A): Annual day-evening-night A-weighted equivalent continuous noise level
LUR: Land-use regression
MS: Multiple Sclerosis
MSP: Medical Services Plan
NAD: Non-Alzheimer’s dementia
NAPS: National Air Pollution Surveillance
NDVI: Normalized Difference Vegetation Index
NO: Nitric oxide
NO_2_: Nitrogen dioxide
OR: Odds ratio
PD: Parkinson’s disease
PM_2.5_: Fine particulate matter
