# Supplementary material for: Road proximity, air pollution, noise, green space and neurologic disease incidence: a population-based cohort study
Source: Environ Health. 2020 Jan 21;19:8. doi: 10.1186/s12940-020-0565-4 (PMC6974975; doi:10.1186/s12940-020-0565-4)
Supplement: Supplementary file 1 — Additional file 1: Table S1. Correlations between road proximity, air pollution, noise and greenness in cohorts of Non-Alzheimer’s dementia, Parkinson’s disease, Alzheimer’s disease and Multiple sclerosis. Table S2. Distributions of exposures from Canadian Urban Environmental Health Research Consortium (CANUE). Table S3. Hazard ratios between exposures and Non-Alzheimer’s dementia stratified by sex (Males, Females), ethnicity (Chinese, South Asian, Visible Minority) and age (> = 65 years, < 65 years). Table S4. Hazard ratios between exposures and Parkinson’s disease stratified by sex (Males, Females), ethnicity (Chinese, South Asian, Visible Minority) and age (> = 65 years, < 65 years). Table S5. Odds ratios between exposures and Alzheimer’s disease and Multiple sclerosis stratified by ethnicity (Chinese, South Asian, Visible Minority). Figure S1. Hazard ratios (95% confidence interval) associated with road proximity for non-Alzheimer’s disease and Parkinson’s disease. HW = Highway, MR = Major road. Figure S2. Odds ratios (95% confidence interval) associated with road proximity for Alzheimer’s disease and Multiple sclerosis. HW = Highway, MR = Major road. Figure S3. Odds ratios (95% confidence interval) associated with air pollution, noise and greenness (per Interquartile range as indicated in Table 3) for Alzheimer’s disease and Multiple sclerosis. PM2.5 = fine particulate matter, NO2 = Nitrogen dioxide, NO = Nitric oxide. [file 12940_2020_565_MOESM1_ESM.docx]

Table S1 Correlations between road proximity, air pollution, noise and greenness in cohorts of Non-Alzheimer’s dementia, Parkinson’s disease, Alzheimer’s disease and Multiple sclerosis

| Non-Alzheimer’s dementia |  | HW50 meters | HW150 meters | MR50 meters | MR50 or HW150 meters | PM_2.5_ | BC | NO_2_ | NO | Noise | Greenness | PM_2.5_^†^ | NO_2_^†^ | Greenness^†^ |
| --- | --- | --- | --- | --- | --- | --- | --- | --- | --- | --- | --- | --- | --- | --- |
|  | HW50 meters | 1 | 0.591 | 0.002 | 0.371 | 0.030 | 0.271 | 0.089 | 0.177 | 0.166 | -0.114 | 0.041 | 0.020 | -0.011 |
|  | HW150 meters |  | 1 | 0.001 | 0.627 | 0.062 | 0.280 | 0.135 | 0.215 | 0.199 | -0.171 | 0.005 | 0.021 | -0.001 |
|  | MR50 meters |  |  | 1 | 0.730 | 0.032 | 0.394 | 0.097 | 0.304 | 0.170 | -0.093 | 0.072 | 0.023 | -0.020 |
|  | MR50 or HW150 meters |  |  |  | 1 | 0.066 | 0.491 | 0.158 | 0.358 | 0.258 | -0.174 | 0.047 | 0.028 | -0.014 |
|  | PM_2.5_ |  |  |  |  | 1 | 0.115 | 0.523 | 0.299 | 0.157 | -0.381 | 0.294 | 0.115 | -0.015 |
|  | BC |  |  |  |  |  | 1 | 0.254 | 0.520 | 0.373 | -0.298 | 0.117 | 0.058 | -0.033 |
|  | NO_2_ |  |  |  |  |  |  | 1 | 0.533 | 0.221 | -0.480 | 0.316 | 0.271 | -0.044 |
|  | NO |  |  |  |  |  |  |  | 1 | 0.399 | -0.481 | 0.284 | 0.252 | -0.055 |
|  | Noise |  |  |  |  |  |  |  |  | 1 | -0.213 | 0.182 | 0.137 | -0.008 |
|  | Greenness |  |  |  |  |  |  |  |  |  | 1 | -0.432 | -0.281 | 0.052 |
|  | PM_2.5_^†^ |  |  |  |  |  |  |  |  |  |  | 1 | 0.371 | -0.132 |
|  | NO_2_^†^ |  |  |  |  |  |  |  |  |  |  |  | 1 | -0.389 |
|  | Greenness^†^ |  |  |  |  |  |  |  |  |  |  |  |  | 1 |
|  |  |  |  |  |  |  |  |  |  |  |  |  |  |  |
| Parkinson’s disease |  | HW50 meters | HW150 meters | MR50 meters | MR50 or HW150 meters | PM_2.5_ | BC | NO_2_ | NO | Noise | Greenness | PM_2.5_^†^ | NO_2_^†^ | Greenness^†^ |
|  | HW50 meters | 1 | 0.590 | 0.003 | 0.370 | 0.029 | 0.270 | 0.089 | 0.178 | 0.167 | -0.115 | 0.042 | 0.021 | -0.011 |
|  | HW150 meters |  | 1 | 0.001 | 0.628 | 0.062 | 0.281 | 0.135 | 0.214 | 0.200 | -0.170 | 0.006 | 0.021 | -0.001 |
|  | MR50 meters |  |  | 1 | 0.731 | 0.033 | 0.393 | 0.098 | 0.304 | 0.170 | -0.094 | 0.073 | 0.024 | -0.021 |
|  | MR50 or HW150 meters |  |  |  | 1 | 0.067 | 0.483 | 0.158 | 0.357 | 0.259 | -0.175 | 0.048 | 0.029 | -0.014 |
|  | PM_2.5_ |  |  |  |  | 1 | 0.114 | 0.524 | 0.298 | 0.158 | -0.380 | 0.295 | 0.116 | -0.016 |
|  | BC |  |  |  |  |  | 1 | 0.255 | 0.520 | 0.373 | -0.299 | 0.116 | 0.059 | -0.033 |
|  | NO_2_ |  |  |  |  |  |  | 1 | 0.534 | 0.220 | -0.480 | 0.316 | 0.272 | -0.044 |
|  | NO |  |  |  |  |  |  |  | 1 | 0.400 | -0.482 | 0.284 | 0.252 | -0.056 |
|  | Noise |  |  |  |  |  |  |  |  | 1 | -0.214 | 0.182 | 0.137 | -0.008 |
|  | Greenness |  |  |  |  |  |  |  |  |  | 1 | -0.433 | -0.282 | 0.053 |
|  | PM_2.5_^†^ |  |  |  |  |  |  |  |  |  |  | 1 | 0.372 | -0.132 |
|  | NO_2_^†^ |  |  |  |  |  |  |  |  |  |  |  | 1 | -0.390 |
|  | Greenness^†^ |  |  |  |  |  |  |  |  |  |  |  |  | 1 |
|  |  |  |  |  |  |  |  |  |  |  |  |  |  |  |
| Alzheimer’s disease |  | HW50 meters | HW150 meters | MR50 meters | MR50 or HW150 meters | PM_2.5_ | BC | NO_2_ | NO | Noise | Greenness | PM_2.5_^†^ | NO_2_^†^ | Greenness^†^ |
|  | HW50 meters | 1 | 0.596 | 0.034 | 0.364 | 0.038 | 0.231 | 0.059 | 0.137 | 0.143 | -0.099 | 0.038 | 0.012 | -0.026 |
|  | HW150 meters |  | 1 | 0.039 | 0.611 | 0.074 | 0.211 | 0.082 | 0.155 | 0.190 | -0.138 | 0.027 | 0.019 | -0.001 |
|  | MR50 meters |  |  | 1 | 0.730 | 0.012 | 0.360 | 0.077 | 0.297 | 0.144 | -0.056 | 0.071 | 0.021 | -0.011 |
|  | MR50 or HW150 meters |  |  |  | 1 | 0.062 | 0.427 | 0.122 | 0.341 | 0.239 | -0.137 | 0.039 | 0.028 | -0.011 |
|  | PM_2.5_ |  |  |  |  | 1 | 0.074 | 0.495 | 0.243 | 0.115 | -0.384 | 0.266 | 0.155 | -0.041 |
|  | BC |  |  |  |  |  | 1 | 0.192 | 0.518 | 0.302 | -0.267 | 0.060 | 0.052 | -0.032 |
|  | NO_2_ |  |  |  |  |  |  | 1 | 0.502 | 0.156 | -0.466 | 0.297 | 0.278 | -0.045 |
|  | NO |  |  |  |  |  |  |  | 1 | 0.327 | -0.446 | 0.239 | 0.237 | -0.045 |
|  | Noise |  |  |  |  |  |  |  |  | 1 | -0.160 | 0.149 | 0.135 | -0.001 |
|  | Greenness |  |  |  |  |  |  |  |  |  | 1 | -0.419 | -0.267 | 0.058 |
|  | PM_2.5_^†^ |  |  |  |  |  |  |  |  |  |  | 1 | 0.359 | -0.132 |
|  | NO_2_^†^ |  |  |  |  |  |  |  |  |  |  |  | 1 | -0.395 |
|  | Greenness^†^ |  |  |  |  |  |  |  |  |  |  |  |  | 1 |
|  |  |  |  |  |  |  |  |  |  |  |  |  |  |  |
| Multiple sclerosis |  | HW50 meters | HW150 meters | MR50 meters | MR50 or HW150 meters | PM_2.5_ | BC | NO_2_ | NO | Noise | Greenness | PM_2.5_^†^ | NO_2_^†^ | Greenness^†^ |
|  | HW50 meters | 1 | 0.587 | 0.013 | 0.366 | 0.061 | 0.268 | 0.083 | 0.191 | 0.170 | -0.136 | 0.007 | 0.002 | -0.025 |
|  | HW150 meters |  | 1 | 0.001 | 0.624 | 0.076 | 0.305 | 0.149 | 0.251 | 0.238 | -0.199 | 0.011 | 0.033 | -0.009 |
|  | MR50 meters |  |  | 1 | 0.733 | 0.004 | 0.411 | 0.131 | 0.344 | 0.172 | -0.106 | 0.074 | 0.056 | -0.043 |
|  | MR50 or HW150 meters |  |  |  | 1 | 0.052 | 0.496 | 0.187 | 0.408 | 0.285 | -0.192 | 0.042 | 0.062 | -0.028 |
|  | PM_2.5_ |  |  |  |  | 1 | 0.124 | 0.483 | 0.323 | 0.161 | -0.386 | 0.321 | 0.137 | -0.024 |
|  | BC |  |  |  |  |  | 1 | 0.269 | 0.544 | 0.374 | -0.321 | 0.145 | 0.111 | -0.031 |
|  | NO_2_ |  |  |  |  |  |  | 1 | 0.552 | 0.214 | -0.480 | 0.356 | 0.323 | -0.038 |
|  | NO |  |  |  |  |  |  |  | 1 | 0.434 | -0.490 | 0.316 | 0.283 | -0.066 |
|  | Noise |  |  |  |  |  |  |  |  | 1 | -0.260 | 0.181 | 0.144 | -0.007 |
|  | Greenness |  |  |  |  |  |  |  |  |  | 1 | -0.442 | -0.290 | 0.045 |
|  | PM_2.5_^†^ |  |  |  |  |  |  |  |  |  |  | 1 | 0.364 | -0.121 |
|  | NO_2_^†^ |  |  |  |  |  |  |  |  |  |  |  | 1 | -0.388 |
|  | Greenness^†^ |  |  |  |  |  |  |  |  |  |  |  |  | 1 |

Note: HW = Highway; MR = Major road; PM = Particulate matter; BC = Black carbon; NO_2_ = Nitrogen dioxide; NO = Nitrogen oxide;

^†^Exposures from Canadian Urban Environmental Health Research Consortium (CANUE)

Table S2 Distributions of exposures from Canadian Urban Environmental Health Research Consortium (CANUE)

| CANUE exposures |  | Minimum | Medium | Maximum | Interquartile range |
| --- | --- | --- | --- | --- | --- |
|  |  |  |  |  |  |
| Non-Alzheimer’s dementia |  |  |  |  |  |
| PM_2.5_ (μg/m³) |  | 0.90 | 7.46 | 13.26 | 2.33 |
| NO_2_ (ppb) |  | 0.10 | 19.26 | 60.42 | 10.04 |
| Greenness |  | -0.10 | 0.34 | 0.77 | 0.15 |
|  |  |  |  |  |  |
| Parkinson’s disease |  |  |  |  |  |
| PM_2.5_ (μg/m³) |  | 0.90 | 7.47 | 13.27 | 2.34 |
| NO_2_ (ppb) |  | 0.10 | 19.27 | 60.42 | 10.03 |
| Greenness |  | -0.11 | 0.34 | 0.78 | 0.16 |
|  |  |  |  |  |  |
| Alzheimer’s disease |  |  |  |  |  |
| PM_2.5_ (μg/m³) |  | 2.10 | 7.53 | 12.60 | 3.30 |
| NO_2_ (ppb) |  | 0.10 | 19.41 | 55.98 | 10.28 |
| Greenness |  | -0.05 | 0.34 | 0.75 | 0.15 |
|  |  |  |  |  |  |
| Multiple sclerosis |  |  |  |  |  |
| PM_2.5_ (μg/m³) |  | 2.30 | 7.40 | 12.60 | 2.33 |
| NO_2_ (ppb) |  | 0.10 | 19.01 | 54.66 | 9.79 |
| Greenness |  | -0.05 | 0.34 | 0.76 | 0.14 |

Table S3 Hazard ratios between exposures and Non-Alzheimer’s dementia stratified by sex (Males, Females), ethnicity (Chinese, South Asian, Visible Minority) and age (>=65 years, <65 years)

| Exposures |  | Non-Alzheimer’s dementia | | | | | | | | |
| --- | --- | --- | --- | --- | --- | --- | --- | --- | --- | --- |
|  |  | Hazard ratio (95% CI)  per Interquartile Range  [IQR] | | | | | | | | |
| Road proximity |  | Non-stratified | Males | Females | Chinese^†^ | South Asian^†^ | Visible minority^†^ | Age (>=65 years) | Age (<65 years) |  |
| Highway <50m |  | 1.03 (0.91, 1.16) | 1.07 (0.88, 1.29) | 1.02 (0.86, 1.21) | 1.13 (0.94, 1.35) | 1.11 (0.93, 1.32) | 1.07 (0.82, 1.38) | 1.02 (0.89, 1.16) | 0.98 (0.68, 1.41) |  |
| Highway <50m + Greenness |  | 1.02 (0.87, 1.20) | 1.06 (0.84, 1.35) | 1.03 (0.84, 1.28) | 1.05 (0.84, 1.31) | 1.10 (0.89, 1.37) | 1.01 (0.73, 1.39) | 1.00 (0.84, 1.19) | 0.96 (0.70, 1.30) |  |
|  |  |  |  |  |  |  |  |  |  |  |
| Highway <150m |  | 1.10 (1.02, 1.19) | 1.11 (0.98, 1.25) | 1.09 (0.99, 1.21) | 1.13 (1.04, 1.30) | 1.12 (1.00, 1.25) | 1.17 (0.99, 1.38) | 1.09 (1.01, 1.19) | 0.97 (0.80, 1.18) |  |
| Highway <150m + Greenness |  | 1.06 (0.96, 1.17) | 1.07 (0.92, 1.24) | 1.04 (0.91, 1.18) | 1.05 (0.92, 1.20) | 1.04 (0.91, 1.19) | 1.07 (0.88, 1.31) | 1.03 (0.92, 1.15) | 0.96 (0.76, 1.21) |  |
|  |  |  |  |  |  |  |  |  |  |  |
| Major road <50m |  | 1.15 (1.08, 1.23) | 1.14 (1.03, 1.28) | 1.17 (1.06, 1.30) | 1.22 (1.09, 1.36) | 1.19 (1.07, 1.33) | 1.23 (1.07, 1.41) | 1.13 (1.05, 1.21) | 1.36 (1.16, 1.62) |  |
| Major road <50m + Greenness |  | 1.15 (1.07, 1.24) | 1.09 (0.96, 1.24) | 1.16 (1.06, 1.26) | 1.19 (1.08, 1.32) | 1.17 (1.06, 1.29) | 1.20 (1.03, 1.40) | 1.11 (1.02, 1.20) | 1.33 (1.14, 1.57) |  |
|  |  |  |  |  |  |  |  |  |  |  |
| Major road <50m or Highway <150m |  | 1.14 (1.07, 1.20) | 1.14 (1.04, 1.25) | 1.13 (1.05, 1.21) | 1.18 (1.09, 1.29) | 1.16 (1.07, 1.26) | 1.21 (1.07, 1.36) | 1.12 (1.05, 1.19) | 1.19 (1.01, 1.41) |  |
| Major road <50m or Highway <150m + Greenness |  | 1.12 (1.05, 1.20) | 1.09 (0.98, 1.22) | 1.13 (1.02, 1.23) | 1.15 (1.05, 1.27) | 1.14 (1.04, 1.26) | 1.15 (0.99, 1.32) | 1.09 (1.01, 1.18) | 1.18 (1.02, 1.36) |  |
| Air pollution |  |  |  |  |  |  |  |  |  |  |
| PM_2.5_ (μg/m³) |  | 1.02 (0.98, 1.05)  [1.54] | 0.98 (0.94, 1.04)  [1.62] | 1.04 (0.99, 1.09)  [1.63] | 1.00 (0.96, 1.05)  [1.54] | 1.03 (0.99, 1.08)  [1.46] | 1.02 (0.95, 1.10)  [1.62] | 1.03 (0.99, 1.06)  [1.56] | 1.05 (0.94, 1.17)  [1.63] |  |
| PM_2.5_ (μg/m³) + Noise |  | 1.02 (0.98, 1.05) | 0.98 (0.93, 1.04) | 1.04 (0.99, 1.09) | 1.01 (0.96, 1.05) | 1.03 (0.99, 1.08) | 1.02 (0.95, 1.10) | 1.03 (0.99 1.06) | 1.05 (0.93, 1.18) |  |
| PM_2.5_ (μg/m³) + Greenness |  | 1.02 (0.98, 1.05) | 0.98 (0.93, 1.03) | 1.04 (0.99, 1.09) | 1.01 (0.96, 1.05) | 1.03 (0.98, 1.07) | 1.01 (0.94, 1.09) | 1.03 (0.99, 1.06) | 1.03 (0.92, 1.15) |  |
| Black carbon (μg/m³) |  | 1.01 (0.98, 1.04)  [1.06] | 1.01 (0.96, 1.05)  [0.97] | 1.02 (0.97, 1.06)  [0.96] | 1.01 (0.97, 1.05)  [0.96] | 1.03 (0.99, 1.08)  [0.97] | 1.08 (1.02, 1.15)  [0.96] | 1.00 (0.96, 1.04)  [1.18] | 1.11 (1.03, 1.20)  [0.90] |  |
| Black carbon (μg/m³) + Noise |  | 1.01 (0.98, 1.04) | 1.00 (0.94, 1.04) | 1.03 (0.98, 1.07) | 1.01 (0.96,1.05) | 1.05 (1.00, 1.10) | 1.09 (1.03, 1.17) | 1.01 (0.96, 1.05) | 1.11 (1.02, 1.23) |  |
| Black carbon (μg/m³) + Greenness |  | 1.01 (0.98, 1.04) | 1.00 (0.96,1.05) | 1.02 (0.97, 1.05) | 1.01 (0.97, 1.05) | 1.02 (0.98, 1.07) | 1.07 (1.01, 1.14) | 1.00 (0.96, 1.04) | 1.11 (1.03, 1.21) |  |
| NO_2_ (ppb) |  | 1.02 (0.99, 1.06)  [9.06] | 1.00 (0.94, 1.06)  [9.03] | 1.05 (0.99, 1.10)  [9.02] | 1.02 (0.98, 1.07)  [8.07] | 1.05 (1.00, 1.10)  [8.59] | 1.08 (1.00, 1.17)  [9.16] | 1.06 (1.02, 1.10)  [9.34] | 1.06 (0.95, 1.19)  [8.89] |  |
| NO_2_ (ppb) + Noise |  | 1.01 (0.97, 1.06) | 0.98 (0.91, 1.04) | 1.04 (0.98, 1.10) | 1.02 (0.96, 1.06) | 1.05 (0.99, 1.11) | 1.06 (0.97, 1.16) | 1.04 (0.99, 1.09) | 1.05 (0.97,1.15) |  |
| NO_2_ (ppb) + Greenness |  | 1.02 (0.99, 1.06) | 0.99 (0.94, 1.06) | 1.05 (0.99, 1.10) | 1.02 (0.97, 1.07) | 1.04 (0.99, 1.10) | 1.05 (0.97, 1.15) | 1.06 (1.01, 1.10) | 1.05 (0.93, 1.19) |  |
| NO (ppb) |  | 1.00 (0.96, 1.04)  [13.47] | 0.99 (0.93,1.05)  [13.50] | 1.01 (0.96, 1.06)  [13.51] | 0.99 (0.94, 1.05)  [13.85] | 1.01 (0.96, 1.07)  [13.13] | 1.08 (1.01, 1.17)  [13.86] | 1.00 (0.95, 1.04)  [13.73] | 1.08 (0.97, 1.21)  [13.53] |  |
| NO (ppb) + Noise |  | 0.99 (0.95, 1.04) | 0.96 (0.90, 1.03) | 1.02 (0.95, 1.08) | 0.98 (0.92, 1.04) | 1.03 (0.97, 1.10) | 1.09 (0.99, 1.19) | 0.99 (0.94, 1.04) | 1.01 (0.98, 1.06) |  |
| NO (ppb) + Greenness |  | 0.99 (0.95, 1.04) | 0.98 (0.91, 1.04) | 1.01 (0.95, 1.07) | 0.99 (0.94, 1.06) | 1.00 (0.94, 1.06) | 1.06 (0.97, 1.15) | 0.98 (0.94, 1.04) | 1.08 (0.96,1.20) |  |
| Noise (L_den_ dB(A)) |  | 1.01 (0.99, 1.04)  [5.53] | 1.01 (0.98, 1.05)  [5.57] | 1.01 (0.98,1.04)  [5.58] | 1.02 (0.99, 1.05)  [5.46] | 1.08 (1.02, 1.14)  [5.40] | 1.04 (0.99, 1.08)  [5.64] | 1.01 (0.98, 1.03)  [5.90] | 1.03 (0.97, 1.10)  [5.40] |  |
| Greenness (NDVI) |  | 0.95 (0.92, 0.97)  [0.11] | 0.94 (0.90, 0.98)  [0.12] | 0.95 (0.91, 0.98)  [0.11] | 0.94 (0.90, 0.97)  [0.11] | 0.99 (0.96, 1.02)  [0.11] | 0.88 (0.82, 0.93)  [0.12] | 0.93 (0.90, 0.96)  [0.11] | 0.91 (0.84, 0.98)  [0.12] |  |

*Covariates included for Non-Alzheimer’s dementia in non-stratified models: Age, sex, comorbidities, household income, education and ethnicity

^†^Chinese (> 10% of population in Neighborhood), South Asian (> 10% of population in Neighborhood), Visible minority (> 10% of population in Neighborhood)

Table S4 Hazard ratios between exposures and Parkinson’s disease stratified by sex (Males, Females), ethnicity (Chinese, South Asian, Visible Minority) and age (>=65 years, <65 years)

| Exposures |  | Parkinson’s disease | | | | | | | | |
| --- | --- | --- | --- | --- | --- | --- | --- | --- | --- | --- |
|  |  | Hazard ratio (95% CI)  per Interquartile Range  [IQR] | | | | | | | | |
| Road proximity |  | Non-stratified | Males | Females | Chinese^†^ | South Asian^†^ | Visible minority^†^ | Age (>=65 years) | Age (<65 years) |  |
| Highway <50m |  | 1.12 (0.91, 1.38) | 1.17 (0.89, 1.53) | 1.09 (0.74, 1.64) | 1.08 (0.74, 1.60) | 1.21 (0.91, 1.61) | 0.97 (0.60, 1.55) | 0.99 (0.77, 1.22) | 1.38 (0.97, 1.98) |  |
| Highway <50m + Greenness |  | 1.12 (0.85, 1.46) | 1.13 (0.78, (1.61) | 1.05 (0.77, 1.43) | 1.01 (0.72, 1.40) | 1.16 (0.82, 1.66) | 0.89 (0.48, 1.63) | 0.97 (0.69, 1.36) | 1.32 (0.84, 2.09) |  |
|  |  |  |  |  |  |  |  |  |  |  |
| Highway <150m |  | 1.06 (0.93, 1.22) | 1.13 (0.94, 1.35) | 0.98 (0.79, 1.20) | 1.02 (0.83, 1.26) | 1.04 (0.86, 1.27) | 1.03 (0.72, 1.47) | 0.96 (0.81, 1.13) | 1.27 (0.99, 1.63) |  |
| Highway <150m + Greenness |  | 1.02 (0.86, 1.21) | 1.08 (0.85, 1.36) | 0.95 (0.73, 1.23) | 1.01 (0.81, 1.30) | 1.03 (0.82, 1.31) | 0.98 (0.72, 1.33) | 0.88 (0.71, 1.09) | 1.27 (0.94, 1.71) |  |
|  |  |  |  |  |  |  |  |  |  |  |
| Major road <50m |  | 1.09 (0.96, 1.23) | 1.05 (0.88, 1.24) | 1.13 (0.98, 1.34) | 0.98 (0.82, 1.18) | 1.18 (0.98, 1.42) | 1.29 (0.98, 1.71) | 1.04 (0.91, 1.20) | 1.24 (0.98, 1.56) |  |
| Major road <50m + Greenness |  | 1.02 (0.89, 1.18) | 1.00 (0.81, 1.21) | 1.05 (0.86, 1.28) | 0.91 (0.74, 1.12) | 1.15 (0.97, 1.35) | 1.21 (0.95, 1.56) | 0.95 (0.80, 1.12) | 1.24 (0.96, 1.59) |  |
|  |  |  |  |  |  |  |  |  |  |  |
| Major road <50m or Highway <150m |  | 1.07 (0.96, 1.18) | 1.11 (0.96, 1.27) | 1.02 (0.88, 1.18) | 0.97 (0.82, 1.12) | 1.13 (0.96, 1.33) | 1.17 (0.91, 1.52) | 1.01 (0.89, 1.14) | 1.22 (1.01, 1.48) |  |
| Major road <50m or Highway <150m + Greenness |  | 0.99 (0.88, 1.13) | 1.04 (0.87, 1.23) | 0.96 (0.80, 1.14) | 0.92 (0.77, 1.10) | 1.11 (0.96, 1.28) | 1.09 (0.88, 1.36) | 0.93 (0.80, 1.07) | 1.18 (0.95, 1.49) |  |
| Air pollution |  |  |  |  |  |  |  |  |  |  |
| PM_2.5_ (μg/m³) |  | 1.09 (1.02, 1.16)  [1.65] | 1.07 (0.98, 1.16)  [1.60] | 1.11 (1.00, 1.21)  [1.63] | 1.01 (0.93, 1.10)  [1.55] | 1.08 (1.01, 1.17)  [1.44] | 1.01 (0.87, 1.15)  [1.60] | 1.09 (1.02, 1.17)  [1.55] | 1.10 (0.95, 1.28)  [1.62] |  |
| PM_2.5_ (μg/m³) + Noise |  | 1.08 (1.01, 1.15) | 1.05 (0.97, 1.15) | 1.10 (0.99, 1.22) | 1.01 (0.92, 1.09) | 1.08 (0.99, 1.16) | 0.98 (0.85, 1.13) | 1.08 (1.01, 1.17) | 1.07 (0.91, 1.25) |  |
| PM_2.5_ (μg/m³) + Greenness |  | 1.08 (1.01, 1.15) | 1.05 (0.96, 1.15) | 1.10 (1.00, 1.21) | 1.00 (0.92, 1.09) | 1.07 (0.99, 1.17) | 0.97 (0.84, 1.13) | 1.08 (1.01, 1.16) | 1.09 (0.93, 1.28) |  |
| Black carbon (μg/m³) |  | 1.03 (0.97, 1.08)  [1.02] | 1.02 (0.95, 1.11)  [0.96] | 1.03 (0.94, 1.12)  [0.97] | 1.00 (0.92, 1.08)  [0.96] | 0.98 (0.90, 1.06)  [0.98] | 1.00 (0.88, 1.12)  [0.96] | 0.99 (0.92, 1.07)  [1.15] | 1.17 (1.05, 1.31)  [0.91] |  |
| Black carbon (μg/m³) + Noise |  | 1.02 (0.96, 1.09) | 1.03 (0.94, 1.12) | 1.02 (0.93, 1.13) | 0.99 (0.91, 1.08) | 0.96 (0.87, 1.05) | 0.95 (0.83, 1.09) | 1.00 (0.91, 1.08) | 1.15 (1.01, 1.31) |  |
| Black carbon (μg/m³) + Greenness |  | 1.01 (0.96, 1.07) | 1.01 (0.93, 1.09) | 1.01 (0.93, 1.11) | 0.98 (0.90, 1.06) | 0.96 (0.88, 1.05) | 0.95 (0.84, 1.09) | 0.96 (0.89, 1.05) | 1.16 (1.05, 1.32) |  |
| NO_2_ (ppb) |  | 1.12 (1.05, 1.20)  [9.03] | 1.09 (1.00, 1.20)  [8.99] | 1.16 (1.04, 1.28)  [9.03] | 1.07 (0.98, 1.16)  [8.08] | 1.16 (1.07, 1.27)  [8.60] | 1.18 (1.02, 1.36)  [9.14] | 1.14 (1.06, 1.24)  [9.33] | 1.12 (0.96, 1.31)  [8.85] |  |
| NO_2_ (ppb) + Noise |  | 1.09 (1.01, 1.18) | 1.04 (0.93, 1.15) | 1.17 (1.04, 1.31) | 1.03 (0.95, 1.14) | 1.15 (1.00, 1.26) | 1.09 (0.93, 1.28) | 1.12 (1.02, 1.22) | 1.11 (0.93, 1.33) |  |
| NO_2_ (ppb) + Greenness |  | 1.10 (1.03, 1.19) | 1.06 (0.96, 1.17) | 1.15 (1.03, 1.28) | 1.06 (0.97, 1.16) | 1.15 (1.06, 1.27) | 1.14 (0.98, 1.33) | 1.12 (1.04, 1.23) | 1.11 (0.95, 1.34) |  |
| NO (ppb) |  | 1.03 (0.96, 1.11)  [13.59] | 1.02 (0.92, 1.13)  [13.60] | 1.05 (0.94, 1.18)  [13.50] | 1.02 (0.93, 1.13)  [13.84] | 0.99 (0.89, 1.09)  [13.12] | 1.03 (0.87, 1.21)  [13.85] | 1.00 (0.91, 1.09)  [13.72] | 1.17 (1.01, 1.35)  [13.52] |  |
| NO (ppb) + Noise |  | 1.01 (0.93, 1.11) | 1.00 (0.89, 1.12) | 1.03 (0.90, 1.17) | 1.01 (0.90, 1.14) | 0.96 (0.85, 1.09) | 0.96 (0.78, 1.15) | 0.98 (0.88, 1.08) | 1.13 (0.96, 1.35) |  |
| NO (ppb) + Greenness |  | 0.99 (0.92, 1.08) | 0.97 (0.86, 1.08) | 1.03 (0.90, 1.17) | 1.00 (0.89, 1.12) | 0.99 (0.86, 1.07) | 0.94 (0.87, 1.13) | 0.94 (0.85, 1.04) | 1.15 (1.02, 1.41) |  |
| Noise (L_den_ dB(A)) |  | 1.01 (0.97, 1.05)  [5.54] | 0.97 (0.91, 1.01)  [5.50] | 1.06 (1.00, 1.13)  [5.58] | 1.02 (0.98, 1.08)  [5.45] | 1.00 (0.94, 1.05)  [5.41] | 0.99 (0.91, 1.07)  [5.63] | 0.99 (0.94, 1.04)  [5.91] | 1.08 (0.99, 1.17)  [5.39] |  |
| Greenness (NDVI) |  | 0.97 (0.93, 1.01)  [0.11] | 0.96 (0.91, 1.04)  [0.12] | 0.96 (0.89, 1.03)  [0.12] | 0.97 (0.91, 1.04)  [0.11] | 0.99 (0.94, 1.06)  [0.11] | 0.91 (0.82, 1.01)  [0.12] | 0.95 (0.91, 1.01)  [0.11] | 0.76 (0.66, 0.87)  [0.12] |  |

*Covariates included for Parkinson’s disease in non-stratified models: Age, sex, comorbidities, household income, education and ethnicity

^†^Chinese (> 10% of population in Neighborhood), South Asian (> 10% of population in Neighborhood), Visible minority (> 10% of population in Neighborhood)

Table S5 Odds ratios between exposures and Alzheimer’s disease and Multiple sclerosis stratified by ethnicity (Chinese, South Asian, Visible Minority)

| Exposures |  | Alzheimer’s disease | | | |  | Multiple sclerosis | | | |
| --- | --- | --- | --- | --- | --- | --- | --- | --- | --- | --- |
|  |  | Odds ratio (95% CI)  per Interquartile Range  [IQR] | | | |  | Odds ratio (95% CI)  per Interquartile Range  [IQR] | | | |
| Road proximity |  | Non-stratified | Chinese^†^ | South Asian^†^ | Visible minority^†^ |  | Non-stratified | Chinese^†^ | South Asian^†^ | Visible minority^†^ |
| Highway <50m |  | 1.19 (0.74, 1.91) | 0.89 (0.21, 1.71) | 0.92 (0.41, 2.07) | 0.86 (0.26, 5.11) |  | 1.20 (0.69, 2.09) | 1.03 (0.43, 2.48) | 1.16 (0.50, 2.66) | 1.10 (0.30, 4.08) |
| Highway <50m + Greenness |  | 0.85 (0.42, 1.74) | 0.78 (0.42, 1.97) | 0.72 (0.48, 1.07) | 0.74 (0.24, 2.72) |  | 1.09 (0.52, 2.29) | 0.75 (0.26, 2.20) | 1.15 (0.37, 3.62) | 0.86 (0.36, 3.61) |
|  |  |  |  |  |  |  |  |  |  |  |
| Highway <150m |  | 1.03 (0.75, 1.41) | 0.81 (0.43, 1.51) | 1.04 (0.62, 1.75) | 0.85 (0.47, 2.55) |  | 0.97 (0.68, 1.39) | 0.92 (0.49, 1.73) | 0.95 (0.54, 1.67) | 0.91 (0.29, 2.47) |
| Highway <150m + Greenness |  | 0.81 (0.53, 1.24) | 0.66 (0.25, 1.29) | 0.80 (0.41, 1.57) | 0.64 (0.43, 4.72) |  | 1.05 (0.66, 1.67) | 0.75 (0.35, 1.59) | 1.21 (0.61, 2.39) | 1.07 (0.19, 3.58) |
|  |  |  |  |  |  |  |  |  |  |  |
| Major road <50m |  | 1.26 (0.96, 1.64) | 1.23 (0.75, 2.03) | 1.29 (0.84, 2.01) | 1.51 (0.49, 4.72) |  | 1.45 (1.06, 1.97) | 1.65 (0.95, 2.86) | 1.11 (0.67, 1.84) | 2.01 (0.65, 6.31) |
| Major road <50m + Greenness |  | 1.12 (0.81, 1.54) | 1.04 (0.64, 1.69) | 0.96 (0.58, 1.59) | 1.44 (0.37, 5.67) |  | 1.75 (1.19, 2.54) | 1.80 (0.97, 3.35) | 1.45 (0.79, 2.67) | 2.13 (0.56, 8.10) |
|  |  |  |  |  |  |  |  |  |  |  |
| Major road <50m or Highway <150m |  | 1.19 (0.95, 1.49) | 1.04 (0.68, 1.56) | 1.21 (0.83, 1.76) | 0.87 (0.36, 2.10) |  | 1.25 (0.96, 1.63) | 1.41 (0.88, 2.26) | 1.09 (0.71, 1.68) | 1.07 (0.46, 2.48) |
| Major road <50m or Highway <150m + Greenness |  | 1.02 (0.77, 1.36) | 1.03 (0.63, 1.69) | 0.92 (0.58, 1.45) | 0.76 (0.19, 1.78) |  | 1.50 (1.08, 2.08) | 1.40 (0.81, 2.41) | 1.44 (0.84, 2.47) | 1.25 (0.42, 3.78) |
| Air pollution |  |  |  |  |  |  |  |  |  |  |
| PM_2.5_ (μg/m³) |  | 0.90 (0.76, 1.07)  [1.54] | 0.92 (0.68, 1.24)  [1.51] | 0.96 (0.73, 1.28)  [1.37] | 0.90 (0.43, 1.90)  [1.51] |  | 1.25 (0.93, 1.70)  [1.72] | 1.63 (0.90, 2.96) [1.67] | 1.39 (0.83, 2.32)  [1.59] | 1.36 (0.35, 2.32)  [1.68] |
| PM_2.5_ (μg/m³) + Noise |  | 0.91 (0.76, 1.09) | 0.90 (0.65, 1.23) | 1.00 (0.73, 1.37) | 0.95 (0.26, 2.16) |  | 1.40 (0.98, 1.97) | 1.72 (0.97, 3.01) | 1.56 (0.80, 3.06) | 1.45 (0.97, 2.15) |
| PM_2.5_ (μg/m³) + Greenness |  | 0.95 (0.80, 1.13) | 1.11 (0.81, 1.54) | 1.05 (0.78, 1.42) | 1.16 (0.57, 2.89) |  | 1.43 (1.04, 1.97) | 1.82 (1.01, 3.65) | 1.45 (0.86, 2.44) | 1.48 (0.76, 2.39) |
| Black carbon (μg/m³) |  | 1.02 (0.88, 1.18)  [1.09] | 1.07 (0.85, 1.35)  [1.01] | 0.91 (0.71, 1.51)  [1.07] | 0.90 (0.49, 1.66)  [1.33] |  | 0.93 (0.75, 1.15)  [0.90] | 0.98 (0.68, 1.39)  [0.97] | 1.11 (0.72, 1.71)  [0.97] | 0.71 (0.30, 1.66)  [0.78] |
| Black carbon (μg/m³) + Noise |  | 0.91 (0.76, 1.08) | 0.99 (0.74, 1.31) | 0.80 (0.59, 1.09) | 1.09 (0.42, 154) |  | 0.95 (0.74, 1.23) | 0.90 (0.60, 1.35) | 1.26 (0.68, 2.27) | 1.48 (0.22, 2.86) |
| Black carbon (μg/m³) + Greenness |  | 1.05 (0.90, 1.22) | 1.17 (0.91, 1.51) | 0.95 (0.74, 1.20) | 1.01 (0.49, 2.10) |  | 0.97 (0.78, 1.21) | 0.99 (0.69, 1.42) | 1.20 (0.76, 1.87) | 0.84 (0.28, 2.52) |
| NO_2_ (ppb) |  | 0.84 (0.70, 0.99)  [8.96] | 0.72 (0.53, 0.97)  [8.08] | 0.88 (0.66, 1.17)  [8.68] | 0.94 (0.43, 2.09)  [9.09] |  | 1.02 (0.78, 1.44)  [8.70] | 1.21 (0.70, 2.07)  [7.75] | 1.57 (0.78, 3.16)  [8.26] | 1.08 (0.11, 2.37)  [8.53] |
| NO_2_ (ppb) + Noise |  | 0.77 (0.63, 0.95) | 0.73 (0.52, 1.01) | 0.86 (0.62, 1.21) | 0.92 (0.29, 2.93) |  | 1.06 (0.69, 1.62) | 1.15 (0.63, 2.12) | 1.41 (0.56, 3.53) | 0.80 (0.13, 5.10) |
| NO_2_ (ppb) + Greenness |  | 0.87 (0.72, 0.15) | 0.83 (0.61, 1.13) | 0.94 (0.69, 1.27) | 0.96 (0.58, 1.37) |  | 1.16 (0.80, 1.68) | 1.26 (0.72, 2.27) | 1.65 (0.88, 3.30) | 1.22 (0.15, 2.21) |
| NO (ppb) |  | 0.91 (0.77, 1.11)  [13.68] | 0.90 (0.65, 1.23)  [13.59] | 0.88 (0.62, 1.24)  [13.52] | 0.86 (0.40, 1.85)  [13.78] |  | 0.85 (0.62, 1.16)  [13.41] | 1.04 (0.65, 1.58)  [14.04] | 0.96 (0.57, 1.60)  [12.64] | 1.05 (0.82, 1.18)  [13.45] |
| NO (ppb) + Noise |  | 0.84 (0.67, 1.05) | 0.89 (0.61, 1.29) | 0.84 (0.55, 1.28) | 0.83 (0.40, 1.25) |  | 0.88 (0.59, 1.29) | 0.94 (0.54, 1.67) | 0.92 (0.51, 1.66) | 1.02 (0.21, 2.06) |
| NO (ppb) + Greenness |  | 0.98 (0.80, 1.20) | 1.14 (0.80, 1.65) | 0.98 (0.67, 1.43) | 1.12 (0.56, 2.74) |  | 0.93 (0.66, 1.29) | 1.09 (0.67, 1.78) | 1.05 (0.62, 1.76) | 1.07 (0.34, 1.18) |
| Noise (L_den_ dB(A)) |  | 0.99 (0.92, 1.08)  [5.75] | 1.01 (0.89, 1.14)  [5.74] | 1.00 (0.90, 1.12)  [5.80] | 0.98 (0.87, 1.29)  [5.79] |  | 0.92 (0.82, 1.03)  [5.50] | 0.86 (0.73, 1.01)  [5.49] | 0.81 (0.69, 0.96)  [5.49] | 0.97 (0.69, 1.36)  [5.80] |
| Greenness (NDVI) |  | 1.24 (1.13, 1.35)  [0.12] | 1.25 (1.08, 1.43)  [0.11] | 1.23 (1.08, 1.40)  [0.12] | 1.27 (1.01, 1.53)  [0.11] |  | 1.14 (1.00, 1.30)  [0.12] | 1.21 (1.00, 1.45)  [0.12] | 1.11 (0.93, 1.34)  [0.11] | 1.11 (0.80, 1.53)  [0.11] |

*Covariates included for Alzheimer’s disease and Multiple sclerosis in non-stratified models: Comorbidities, household income, education and ethnicity

^†^Chinese (> 10% of population in Neighborhood), South Asian (> 10% of population in Neighborhood), Visible minority (> 10% of population in Neighborhood)

Figure S1 Hazard ratios (95% confidence interval) associated with road proximity for non-Alzheimer’s disease and Parkinson’s disease. HW = Highway, MR = Major road.

**
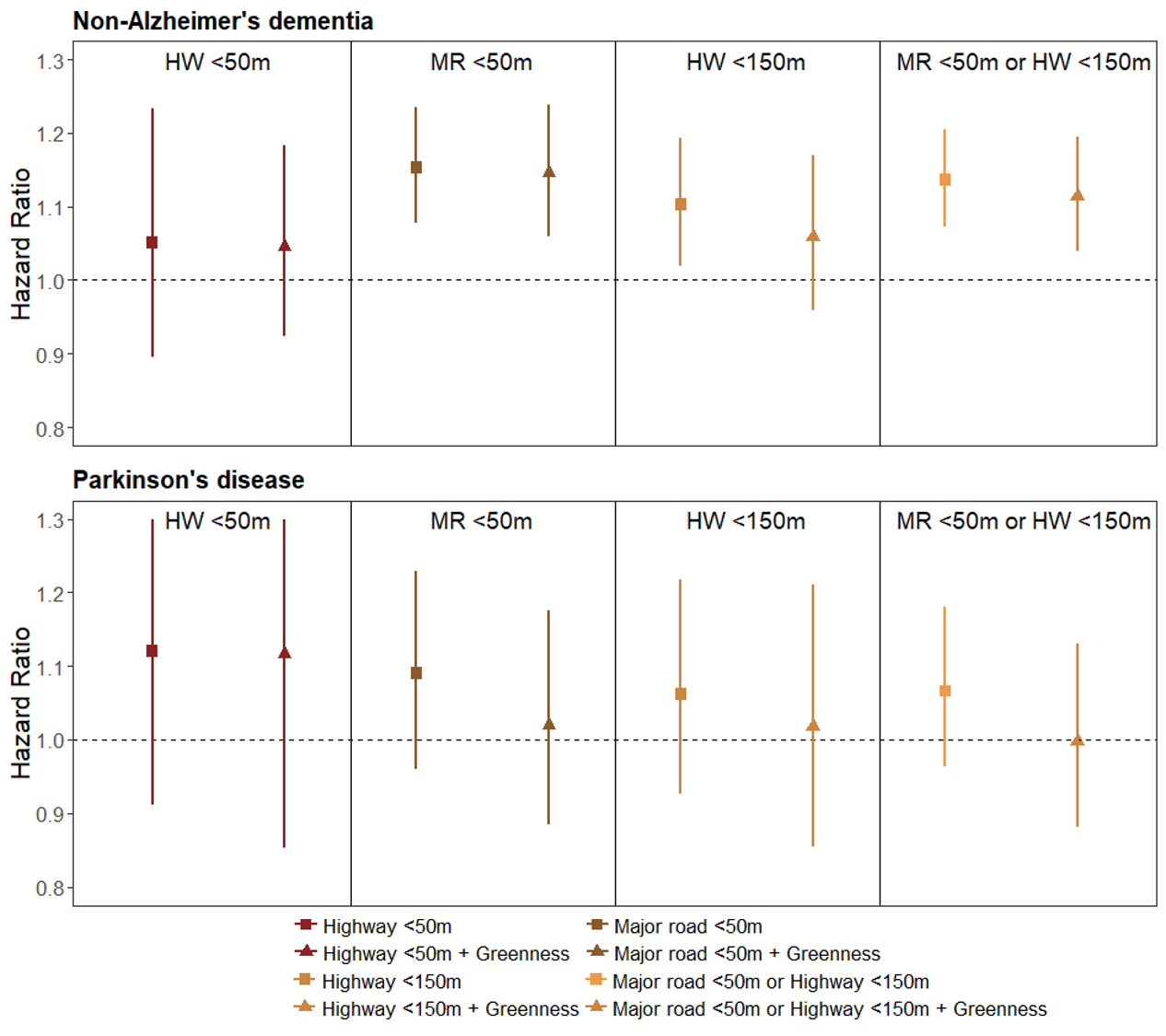
**

Figure S2 Odds ratios (95% confidence interval) associated with road proximity for Alzheimer’s disease and Multiple sclerosis. HW = Highway, MR = Major road.

**
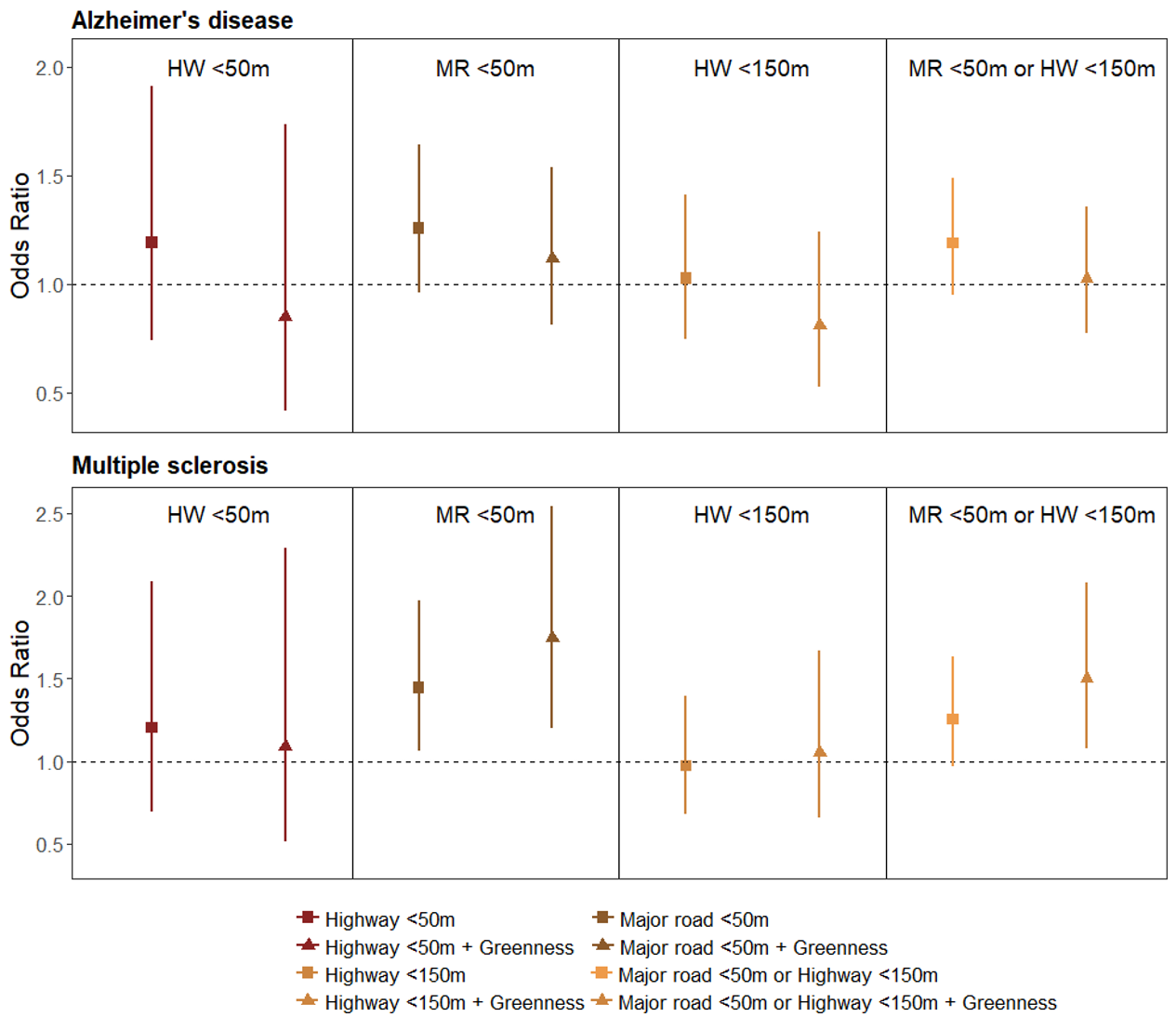
**

Figure S3 Odds ratios (95% confidence interval) associated with air pollution, noise and greenness (per Interquartile range as indicated in Table 3) for Alzheimer’s disease and Multiple sclerosis. PM_2.5_ = fine particulate matter, NO_2_ = Nitrogen dioxide, NO = Nitric oxide.

**
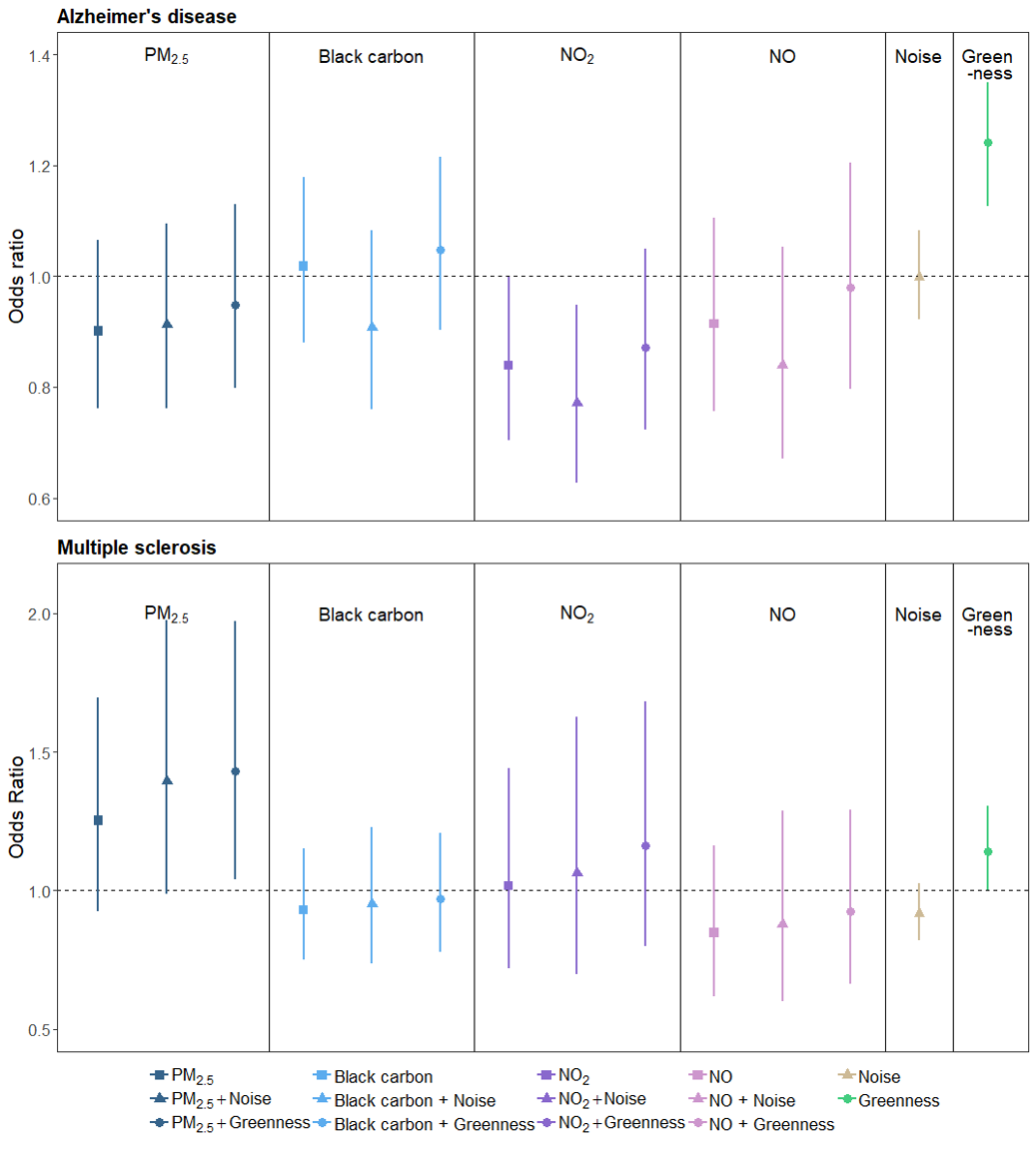
**
